# Assessing animal individuality: links between personality and laterality in pigs

**DOI:** 10.1093/cz/zoy071

**Published:** 2018-09-28

**Authors:** Charlotte Goursot, Sandra Düpjan, Ellen Kanitz, Armin Tuchscherer, Birger Puppe, Lisette M C Leliveld

**Affiliations:** 1 Institute of Behavioural Physiology; 2 Institute of Genetics and Biometry, Leibniz Institute for Farm Animal Biology (FBN), Wilhelm-Stahl-Allee 2, D Dummerstorf, Germany; 3 Behavioural Sciences, Faculty of Agricultural and Environmental Sciences, University of Rostock, Justus-von-Liebig-Weg 6B, Rostock D, Germany

**Keywords:** approach-withdrawal hypothesis, coping, emotion, hemispheric dominance, motor lateralization, temperament

## Abstract

Animal individuality is challenging to explain because individual differences are regulated by multiple selective forces that lead to unique combinations of characteristics. For instance, the study of personality, a core aspect of individuality, may benefit from integrating other factors underlying individual differences, such as lateralized cerebral processing. Indeed, the approach-withdrawal hypothesis (the left hemisphere controls approach behavior, the right hemisphere controls withdrawal behavior), may account for differences in boldness or exploration between left and right hemispheric dominant individuals. To analyze the relationships between personality and laterality we tested 80 male piglets with established laterality patterns for 2 motor functions (tail curling direction and the side of the snout used for manipulation) and a combined classification integrating both motor functions using cluster analysis. We analyzed basal salivary testosterone and cortisol along with their behavior in standardized tests as pre-established indicators of different personality traits (Boldness, Exploration, Activity, Sociability, and Coping). We found that the direction of the single motor biases showed significant associations with few personality traits. However, the combined laterality classification showed more, and more robust, significant associations with different personality traits compared with the single motor biases. These results supported the approach-withdrawal hypothesis because right-biased pigs were bolder and more explorative in a context of novelty. Additionally, right-biased pigs were more sociable than left-biased pigs. Therefore, the present study indicates that personality is indeed related to lateralized cerebral processing and provides insight into the multifactorial nature of individuality.

Within a population, individuals rarely behave uniformly but rather display complex combinations of different strategies in a variable environment. The maintenance of such variation among phenotypes within a species is hypothesized to be regulated by multiple—sometimes conflicting—selective forces (Sih 1992). As a consequence, individuals in the same environment can differ in their trade-offs between such selective forces leading to varying combinations of characteristics regulated at different levels (e.g., genetics, physiology, neurobiology, or behavior) (Sih et al. 2004). For this reason, the basis of individuality is difficult to describe and represents a challenge in various research fields (Pradeu 2016), for instance animal personality research. Personality is multifactorial and can be defined as a correlated set of individual behavioral and physiological traits that are consistent over time and situations (Réale et al. 2007). Several frameworks have been proposed in an effort to classify them (Koolhaas et al. 1999; Dingemanse and Wolf 2010; Finkemeier et al. 2018), such as the 5 traits model: Boldness (also described as Fearfulness), Exploration, Aggressiveness, Activity, and Sociability (Réale et al. 2007). However, because personality represents only one aspect of individuality, other features underlying individual differences such as physiological (Careau et al. 2008) and neurobiological mechanisms (Freund et al. 2013) should be taken into account (Sih et al. 2015) to better comprehend this phenomenon.

One approach to investigating individual variation in neurobiological mechanisms is the study of individual tendencies to use 1 hemisphere of the brain more than the other, resulting in individual hemispheric dominance patterns (left or right) that are observable through contralateral individual side preferences in simple motor tasks (Rogers 2009; see also Rogers et al. 2013; Rogers and Vallortigara 2015; Vallortigara and Versace 2017). Indeed, because each hemisphere controls the contralateral side of the body, it is acknowledged that an individual’s dominance of 1 hemisphere results in motor preferences on the contralateral side (Kinsbourne 1997; Jackson 2008; Wright and Hardie 2015). The 2 hemispheres of the brain specialize in different cognitive processes (referred to as cerebral lateralisation), which is believed to enhance individual cerebral efficiency (Vallortigara 2000, 2006; Ghirlanda and Vallortigara 2004; Rogers et al. 2004; Vallortigara and Rogers 2005). For example, the processing of emotions is lateralized with indications that the right hemisphere controls the negative (or withdrawal) emotions and the left hemisphere controls the positive (or approach) emotions (Davidson 1992; Quaranta et al. 2007; Siniscalchi et al. 2013; see Leliveld et al. 2013 for a discussion of current hypotheses). Due to these different functions of the 2 hemispheres, individual tendencies to use 1 hemisphere more than the other can lead to differences in the response to environmental stimuli (Tops et al. 2017) that are expressed through consistent coping styles or temperaments (Rogers 2009). As a result, studies on different species have found that right-handed individuals are bolder (Hopkins and Bennett 1994; Braccini and Caine 2009), more explorative (Cameron and Rogers 1999; Gordon and Rogers 2010), or more sociable (Westergaard et al. 2003; Gordon and Rogers 2010) than left-handed individuals. Links between the strength of laterality (the degree of dependence on 1 hemisphere) and personality have also been reported (Reddon and Hurd 2009; Found and St. Clair 2017). Such interactions between laterality and personality could also be exploited in studies on animal welfare (Rogers 2010; Leliveld et al. 2013). For this reason, an interest in laterality in farm animals is growing (in sheep: Morgante et al. 2007; Versace et al. 2007; Morgante et al. 2010; in cattle: Phillips et al. 2015; Kappel et al. 2017). However, with the exception of domestic fowl, not much is known about laterality in many farm animals.

Like personality, motor lateralization is suggested to be a multifactorial phenomenon expressed through different lateralization patterns within an individual for different motor functions (Healey et al. 1986; Forrester 2017), which are not necessarily biased in the same direction (Noonan and Axelrod 1989; Mohr et al. 2003). As a consequence, studies in dogs and chimpanzees found that individual motor lateralization patterns differentially affect personality traits (Hopkins and Bard 1993; Batt et al. 2009; Barnard et al. 2017), highlighting the importance of a multifactorial approach. Therefore, in our study we aimed to increase our understanding of the complex nature of individuality through an integrative investigation of links between personality and laterality in pigs *Sus scrofa*.

As a social and generalist species that is one of the most widely distributed mammals in the world (Massei and Genov 2004; Keiter et al. 2016), the pig represents a suitable model for studying individuality. Because its domestication has been characterized by a long history of unintentional human selection (Marshall et al. 2014) and constant gene flow between European wild boars and Asian domestic pigs (Bosse et al. 2014; Frantz et al. 2015), it remains a species with substantial genetic variability (Amills et al. 2010) and a relatively unchanged behavioral repertoire and cognitive abilities (D’Eath and Turner 2009; Puppe et al. 2012). Many personality traits have been studied in pigs such as Boldness (Marchant-Forde 2002; Vetter et al. 2016), Exploration (van Erp van der Kooij et al. 2002; Brown et al. 2009), Activity (Krause et al. 2017), Sociability (Forkman et al. 1995), and Coping (Hessing et al. 1993; Forkman et al. 1995). Recently, the first multifactorial classification of the pigs’ potential individual hemispheric dominance was made by studying their laterality for 2 motor “functions”: manipulation with the snout and tail curling, and combining both functions to identify individuals with consistent biases across both motor functions using a cluster analysis (Goursot et al. 2017).

With this previous knowledge on personality and laterality in pigs, we aimed to uncover possible links between these 2 multifactorial phenomena. To do this, we tested subjects whose individual motor lateralization patterns had been established in Goursot et al. (2017) in a set of personality tests (*Backtest*, *Human Approach Test*, *Open Door Test*, *Open-Field Test*, and *Novel Object Test*) (Forkman et al. 1995; Ruis et al. 2000; van Erp van der Kooij et al. 2002). These tests have been shown to meet the requirements for being used as personality tests in that they correlate with each other and are repeatable over time (*Backtest*: Forkman et al. 1995; Zebunke et al. 2015, 2017; *Human Approach*, *Open Door*, and *Novel Object Tests*: Spoolder et al. 1996; Ruis et al. 2000; van Erp van der Kooij et al. 2002; Brown et al. 2009; *Open-Field Test*: Friel et al. 2016). While most parameters measured in these tests can potentially be linked to more than just 1 personality trait (e.g., latency to contact an unfamiliar object can depend on both Boldness and Exploration), based on the existing body of literature we assigned each parameter to the trait by which it is influenced most (Table 1). For Boldness, we used the latencies to contact a novel human (*Human Approach Test*) or a novel object (*Novel Object Test*), or to enter a new environment (*Open Door Test*; van Erp van der Kooij et al. 2002; Brown et al. 2009), as well as escape attempts and the proportion of high frequency calls in the *Open-Field Test* (Otten et al. 2007; Leliveld et al. 2017). For Exploration we used exploration of an Open Field (*Open-Field Test*), a novel object (*Novel Object Test*), or a novel human (*Human Approach Test*; van Erp van der Kooij et al. 2002; Brown et al. 2009). For Activity we used locomotion in the *Open-Field Test* (Zebunke et al. 2017). We also determined the coping style, tested in a *Backtest*, which is not included in the framework of Réale et al. (2007), but is argued to underlie personality in pigs (Forkman et al. 1995; Finkemeier et al. 2018). Additionally, we performed bioacoustic analyses because vocalizations have been found to provide useful insight into pig personality (Friel et al. 2016; Leliveld et al. 2017). The total number of vocalizations in the *Open-Field Test* was used as an indicator of Sociability, because it reflects the motivation to remain close to conspecifics (Forkman et al. 1995; Koolhaas and van Reenen 2016). Lastly, we determined the testosterone–cortisol ratio from saliva, because the interaction between these hormones has been found to predict any of the following 3 traits in humans: Boldness, Sociability, or Aggressiveness (Terburg et al. 2009; Mehta and Prasad 2015). Using this integrative approach, we studied the relationships between individual lateralization patterns and these pre-established personality traits. For this, we compared the left-biased and the right-biased individuals and predicted to find more substantial differences when considering combined motor lateralization patterns than when considering each motor function alone, because animals with consistent biases across motor functions are supposed to have a stronger hemispheric dominance, and therefore are more likely to show more pronounced, consistent individual behavioral patterns. Such patterns could thus be considered indicative of common neurophysiological mechanisms underlying these 2 aspects of individuality.

**Table 1.** Summary of the parameters measured for each personality trait and by each test: Human Approach Test (HAT), Open Door Test (ODT), Open-Field Test (OFT), Novel Object Test (NOT), Saliva Sampling (S), and the Backtest (BT)

Trait	Test	Parameter
Boldness	HAT	Latency to approach the novel human (front legs <0.5 m from the novel human) (s)
	ODT	Latency to leave the pen (cross the border with the front legs) (s)
	OFT	Proportion of high frequency calls among all analyzed calls (%)
	OFT	Number of escape attempts (jumping/raising the front legs against the wall) (number/session)
	NOT	Latency to touch the Novel Object (s)
	S	Testosterone/cortisol ratio^a^ (mean of the 3 daily ratios)
Exploration	HAT	Duration in proximity of the novel human (front legs <0.5 m from the novel human) (s)
	OFT	Duration of exploring the Open Field (manipulating the floor or walls with the snout) (s)
	OFT	Frequency of exploring the Open Field (manipulating the floor or walls with the snout) (number/session)
	NOT	Duration of touching the Novel Object with the snout (s)
	NOT	Frequency of touching the Novel Object with the snout (number/session)
Activity	OFT	Duration of locomotion (moving with at least 3 feet) (s)
	OFT	Frequency of locomotion (moving with at least 3 feet) (number/session)
Sociability	OFT	Number of vocalizations (during minutes 3 and 4)
	S	Testosterone/cortisol ratio^a^ (mean of the 3 daily ratios)
Aggressiveness	S	Testosterone/cortisol ratio^a^ (mean of the 3 daily ratios)
Coping	BT	Duration of struggling (s)
	BT	Frequency of struggling bouts (number/minute)
	BT	Latency to start struggling (s)

a The ratio T/C may be informative on boldness, sociability, or aggressiveness. Please be aware that each parameter can be influenced by several personality traits; we decided, however, to assign it here to the trait with the largest impact.

## Materials and Methods

### Animals and housing

Details on animals and housing are described in Goursot et al. (2017). In short, the subjects were 80 group-housed pre-pubertal, uncastrated male German Landrace piglets (aged 5*–*7 weeks during the experimental period) studied in 5 consecutive replicates. Before weaning, the subjects were submitted to 4 *Backtests* to determine their coping style (see description below). Healthy subjects that were classified as having an active (high reactive) or passive (low reactive) coping style—according to the criteria from Zebunke et al. (2015)—were preferentially pre-selected for the study.

The subjects were weaned at 28 days (day 0 in our study) and subsequently housed in a group of 20 pigs (1 group per replicate). From days 4–6 post-weaning, the pigs were habituated to the experimental procedures, particularly to handling and the food reward (chocolate raisins) used in the laterality tests. We selected 16 individuals per replicate based on the following criteria (in order of importance): the absence of illness/injuries, eating a food reward when alone, an active or passive coping style (preferred over an intermediate coping style), relatedness to other subjects (the use of full siblings was avoided where possible), and the absence of extreme nervousness when alone in an unfamiliar environment. Each subject was randomly given an ID-number, which determined the individual tests order throughout the entire experiment. During the laterality tests, the subjects were fed an age-appropriate ration once per day; the rest of the time food was available *ad libitum.* Water was available *ad libitum*. The animals had permanent access to a chewing toy, and straw and other rooting materials were provided twice a day for enrichment.

### General procedure

**Figure 1.**
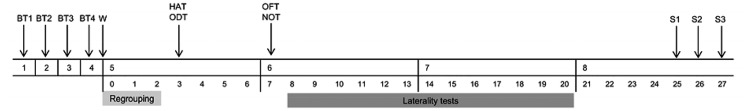
Schedule of the general procedure for each replicate. The numbers in the bottom row indicate the days after weaning (W), whereas the numbers in the top row indicate the weeks of age. BT1-4, Backtest 1–4; HAT, Human Approach Test; ODT, Open Door Test; OFT, Open-Field Test; NOT, Novel Object Test; and S1-3, saliva samples 1–3.

The experimental schedule is shown in Figure 1. The 4 *Backtests* were performed before weaning, whereas the other 4 personality tests were performed after weaning. On the morning of day 4 post-weaning, the *Human Approach Test* and the *Open Door Test* were performed consecutively in the group. On day 7 post-weaning, the subjects were individually subjected to a combined *Open-Field Test* and *Novel Object Test*. The laterality tests were performed from day 8 to day 21 post-weaning. On days 25–27 post-weaning, saliva was sampled (S1, S2, and S3) for analysis of cortisol and testosterone.

### Laterality tests

Details of the experimental procedures for determining individual motor lateralization patterns are published in Goursot et al. (2017). Briefly, we tested lateralized manipulation with the snout and tail curling direction, and determined laterality for both functions as well as a cluster analysis-based classification of individual lateralization patterns across these 2 motor functions. For manipulation with the snout, the subjects were trained to open a flap door to retrieve a food reward and then forced to use either the left (L) or right (R) side of the snout to open it (details in Goursot et al. 2017). For tail observations, we noted the direction of spontaneous curling of the tail: left curling (L, the tip of the tail is situated to the left of the base), right curling (R, the tip of the tail is situated to the right of the base) (details in Goursot et al. 2017). Based on significant individual biases to use one side over the other (tested in a binomial test), individuals were classified as either lateralized (LAT) with a significant bias (L or R) or ambilateral (A, no significant bias) for each motor function. Additionally, for each motor function, a continuous laterality index was calculated as follows: LI =(R − L)/(R + L), where R is the number of right observations and L is the number of left observations. Individual lateralization patterns across the 2 motor functions (combined laterality classification) were determined with a cluster analysis based on the LI of the 2 functions, identifying those subjects who had a consistent lateralization pattern across functions (e.g., RR: right biased for snout use and tail curling direction) and those who were inconsistent (e.g., RL: right biased for snout use, but left biased in tail curling direction; details in Goursot et al. 2017). In this study, we compared only the subjects with consistent biases for both functions (RR vs. LL, see the section “Statistical Analyses”). As described in Goursot et al. (2017), the majority of the subjects were lateralized for snout use (29R and 32L versus 15A subjects) and tail curling (48R and 28L versus 2A subjects). The cluster analysis revealed 18RR subjects (right-biased for both functions), 12LL subjects (left-biased for both functions), and 40 mixed subjects (opposite biases for both functions). The RR subjects and the LL subjects are henceforth referred to as “R-biased pigs” and “L-biased pigs,” respectively.

### Personality tests

The observed parameters are listed in Table 1. Apart from the *Backtest*, all behavioral analyses were made from video recordings using The Observer (The Observer XT 11, Noldus Information Technology bv, The Netherlands), and the data were submitted to an inter-observer reliability test (one of each test was observed by another observer), which resulted in the following kappa indices: 0.89 for the *Open-Field Test*, *Novel Object Test*, and *Human Approach Test*, and 0.99 for the *Open Door Test*. These kappa scores indicate almost perfect agreement between the observations.

#### Backtest

As mentioned previously, we performed 4 *Backtests* at 1-week intervals according to the standardized method of Zebunke et al. (2015). In short, an experimenter put each subject on its back for 1 min and observed its struggling attempts. The mean latency, mean frequency, and mean duration of struggling in the 4 tests were calculated for each individual, because these parameters have been shown to be consistent over time and continuously distributed (Zebunke et al. 2015). The parameters are considered indicators of Coping, whereby a longer duration, higher frequency, and shorter latency indicate a more active coping style.

#### Human Approach Test and Open Door Test

The *Human Approach Test* and the *Open Door Test* were performed in the home pen on the entire group as described by Leliveld et al. (2017) and were recorded using a video camera centrally positioned above the pen. In short, in the *Human Approach Test*, an unfamiliar person wearing unusual clothing entered the pen, positioned themselves against the wall facing the piglets, and stood still for 5 min. The *Open Door Test* was performed immediately after the *Human Approach Test*; the door of the pen onto the corridor was opened for 5 min, enabling the piglets to leave the pen. For the *Human Approach Test*, we scored the latency and the duration of being in proximity to the human (<0.5 m). For the *Open Door Test*, we scored the latency to leave the home pen.

#### Open-Field Test and Novel Object Test

The *Open-Field Test* and the *Novel Object Test* were performed on individual pigs as described in Stracke et al. (2017). In short, these tests occurred in a testing arena (Open Field) located in a sound-attenuated test room. A video camera (connected to a digital video recorder) as well as a microphone (Sennheiser ME64/K6 connected to a Marantz PMD 670 recorder; sampling rate, 44.1 kHz; accuracy, 16 bit; mono) were centrally positioned above the Open Field. Each pig was guided from its home pen into the arena and was left alone. After 5 min, a blue plastic container—the Novel Object—was placed in the center of the Open Field and the animal was observed for another 5 min. The Open Field was cleaned between subjects. For the *Open-Field Test*, we scored the duration and frequency of locomotion and exploration and the frequency of escape attempts. Vocalizations during the *Open-Field Test* were analyzed separately (see below). For the *Novel Object Test*, we scored the latency, frequency, and duration of touching the Novel Object.

### Acoustic analyses

Based on an initial count of all vocalizations from the recordings, the third and fourth minutes of the *Open-Field Test* were identified as the periods of maximum vocal response. Each call produced during these 2 min was analyzed in Avisoft-SAS Lab Pro (Version 5.2.05; Avisoft Bioacoustics, Berlin, Germany) using the same methods and settings as described in Leliveld et al. (2016, 2017). We analyzed a total of 13,198 calls. Of these, 1,979 calls had background noise (typically footsteps). Such calls were included in the total number of vocalizations but were excluded from the acoustic analyses, leaving 11,219 calls. Some calls (*N *=* *4,581) were found to consist of a combination of 2 distinctly different acoustic structures that were easily distinguishable on the oscillogram (mainly grunt-squeals). The different parts of these calls were therefore analyzed separately. The calls were analyzed using the “automatic parameters measurement” option in the spectrogram window (settings: 1,024 FFT length, Hamming window, 50% window overlap, frequency resolution of 43 Hz, temporal resolution of 11.6 ms, and high-pass cut-off frequency at 100 Hz). The following parameters were measured: duration, duration from start to maximum amplitude (% of call duration; DurMax), interval (time from previous call), peak frequency, the minimum and maximum frequency and the resulting bandwidth, 3 quartiles that describe the distribution of energy over the frequency range (Q25, 25%; Q50, 50%; and Q75, 75% of the energy in the frequency range), the number of peaks (above −20 dB, hysteresis: 10 dB), the frequency of the first 2 peaks (F1 and F2), the entropy and the harmonic-to-noise ratio (HNR). The frequency parameters were log-transformed to control for the logarithmic character of animal sound production and sound perception (Cardoso 2013). Call elements were classified in a cluster analysis using the same procedure as Leliveld et al. (2016, 2017). Parameters that did not correlate strongly with other parameters (Spearman rank correlations, |rs|<0.9; CORR procedure in SAS (version 9.4; SAS Institute Inc., Cary, NC, USA)) and that followed a multimodal distribution (determined using the KDE procedure) were selected. Accordingly, 8 parameters (duration, DurMax, peak frequency, F1, F2, HNR, maximum frequency, and Q75) were entered into the FASTCLUS procedure of SAS (maxiter =100, strict =5). To determine the number of clusters that best represented the data, the Cubic Cluster Criterion (CCC) and the Pseudo *F*-statistic were examined.

### Saliva sampling and analyses

Saliva samples were collected prior to any other intrusion between 08:00 and 08:30 h in the morning on 3 consecutive days by allowing the pigs to chew on synthetic swabs (Salivette^®^Cortisol, Sarstedt AG & Co., Germany) for 20–30 s. The swabs were placed in Salivette tubes and centrifuged at 2,500 g for 15 min at 4°C. The saliva samples were stored at −20°C until analysis. After thawing, the samples were spun at 2,500 g for 5 min, resulting in a clear supernatant with low viscosity.

The analysis of cortisol concentrations in the saliva was performed in duplicate in 50 µl samples using a commercial Saliva ELISA kit (Demeditec Diagnostics GmbH, Germany) according to the instructions of the manufacturer. The cross-reactivity of the cortisol antiserum has been measured against various compounds and was 63.4% for prednisolone, 10.4% for 11-deoxycortisol, 5.2% for corticosterone, and less than 0.1% for any further competing steroids. The assay was validated for use with porcine saliva. The sensitivity was 0.08 ng/ml, and the intra- and inter-assay coefficients of variation (CV) were 3.4% and 6.6%, respectively.

The saliva testosterone concentration was analyzed in duplicate in 100 µl samples using the Demeditec Saliva ELISA kit (Demeditec Diagnostics GmbH, Germany) according to the manufacturer’s guidelines. The cross-reactivities of the antibody to 5α-dihydrotestosterone and androstendione were 23.3% and 1.6%, respectively. The lowest level of testosterone that could be detected by this assay in porcine saliva was 8.9 pg/ml, and the intra- and inter-assay CVs were 5.1% and 12.8%, respectively. After the analysis of cortisol (C) and testosterone (T), a daily T/C ratio (based on the 3 daily values per parameter) was calculated for each individual. The mean of these daily ratios was then calculated.

### Statistical analyses

We used SAS version 9.4 for the statistical analyses. We tested the effect of the direction as well as the effect of strength of laterality on the personality traits. The individual parameters were first analyzed using a 2-way analysis of variance (ANOVA; MIXED procedure) with the individual parameters as dependent variables and the motor bias (L or R for tail, snout, or combined), replicate, and their interaction as fixed factors. Parameters that showed significant interaction effects between replicate and motor bias were considered as not reliable enough for our sample and were therefore excluded from further analyses, as such an interaction can indicate that effects are inconsistent across replicates. For the other parameters, the model was reduced to a 2-way ANOVA (MIXED procedure) with the individual parameters as dependent variables and the motor bias and replicate as fixed factors (without their interaction). Because sample sizes differed among parameters (see the “Results” section), the power was calculated for a better comparison. For each significant effect, the power (POWER procedure; significance level alpha = 0.05) was calculated based on group means, residual standard deviations, and sample sizes. Significant results with a power above 0.7 (indicating a 70% chance of reproducing the result if the experiment was repeated) were considered to be robust. In order to analyze effects of strength of laterality irrespective of its direction we compared lateralized (LAT) subjects to ambilateral (A) subjects, but we did not perform an ANOVA for tail curling because only 2 individuals were classified as A for this motor function. Because the combined classification was based on 50% of the LI for tail curling, the strength of laterality was also not analyzed for this classification.

## Results

Our analyses varied in their sample size for several reasons. As described by Goursot et al. (2017), we had different sample sizes per motor function: 29R, 32L, and 15A subjects for snout use; 48R, 28L, and 2A subjects for tail curling; and 18RR and 12LL subjects for the combined classification. In the analysis of the T/C ratio, we included only individuals with all 3 daily T/C ratios available (*N *=* *59). The sample size was also reduced for the *Human Approach Test* (*N*=67) and the *Open Door Test* (*N*=77) due to difficulties in identifying some individuals during the video analysis, although all the animals approached the human or left the pen. The following results revealed significant interactions of replicate and laterality: the mean frequency and the mean latency of struggling during the *Backtest* for tail bias (frequency: *F*_4,__66_ = 3.77, *P *=* *0.008; latency: *F*_4,__66_ =4.68, *P *=* *0.002, *N *=* *76) and for the combined laterality classification (frequency: *F*_3,__21_ = 3.30, *P *=* *0.040; latency: *F*_3,__21_ = 4.64, *P *=* *0.012, *N *=* *30); the T/C ratio (*F*_2,__51_ = 4.16, *P *=* *0.021, *N *=* *59); and the number of vocalizations (*F*_2,__68_ = 3.17, *P *=* *0.048, *N *=* *76) for the strength of snout laterality. These parameters were not taken into account in subsequent analyses with the respective laterality pattern. We will present only significant *F*-test results; a complete list of statistical results can be found in the Supplementary Material.

### Call clusters

**Figure 2.**
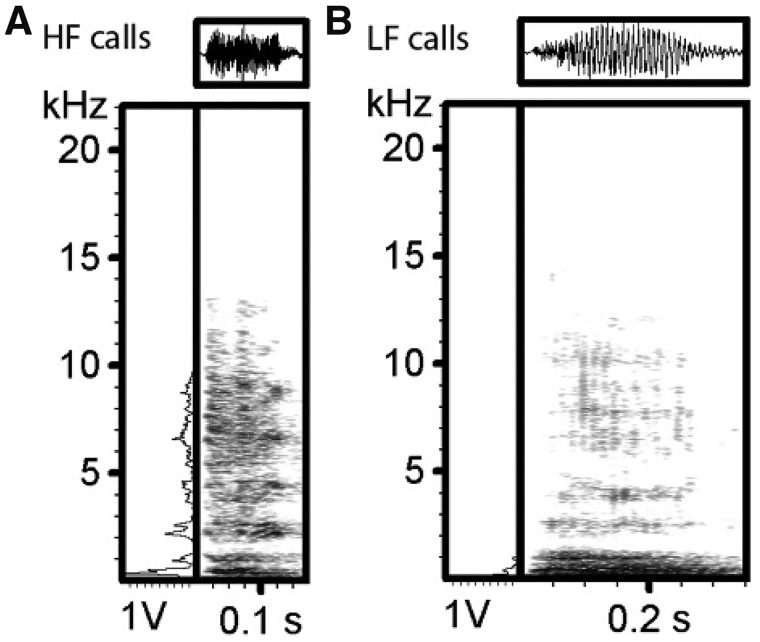
Oscillograms (above), power spectra (left), and spectrograms (right) of typical calls (i.e., close to the cluster center) resulting from a cluster analysis (Pseudo *F *=* *5,798.9, CCC=382,880, *N *=* *11,219) for the high frequency (HF) cluster (*N *=* *5,530) (**A**) and the low frequency (LF) cluster (*N *=* *5,590) (**B**).

Based on the cluster analysis, we found that a 2-cluster option resulted in the highest CCC and Pseudo *F*-statistic values (Pseudo *F *=* *5,798.9, CCC* *=382,880). Based on the differences in the frequency values, these 2 clusters were renamed as high frequency calls (mean peak frequency [Hz]: 1,333.26 ± 1,964.83, *N *=* *5,530) and low frequency calls (mean peak frequency [Hz]: 171.71 ± 106.10, *N *=* *5,590). Ninety-nine calls could not be assigned to either of these clusters. Typical calls for each cluster are shown in Figure 2.

### Effect of single motor biases on personality parameters

**Figure 3.**
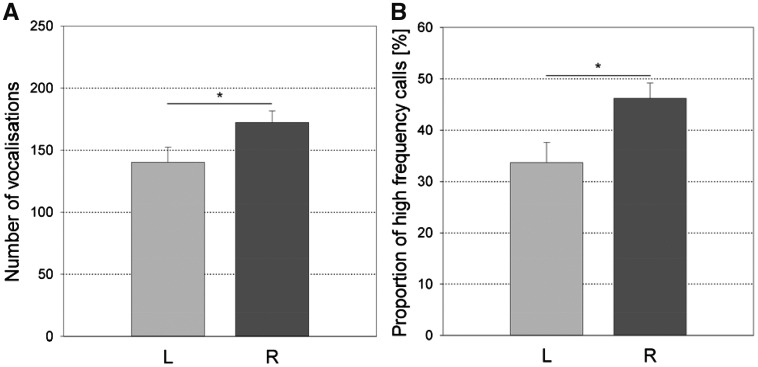
Differences for (**A**) the number of vocalizations in the Open-Field Test (*F*_1,70_=4.19, *P *=* *0.044, *N *=* *76) and (**B**) the proportion of high frequency calls produced in the Open-Field Test (*F*_1,70_=6.14, *P *=* *0.016, *N *=* *76) between left- (L) and right-tailed (R) individuals (least square means and standard errors). *P <* *0.05.

Concerning the direction of tail laterality (Figure 3), the R-tailed individuals vocalized more (*F*_1,__70_ = 4.19, *P* = 0.044, *N* = 76, power < 0.7) and produced a higher proportion of high frequency calls (*F*_1,__70_ = 6.14, *P* = 0.016, *N* = 76, power = 0.719) during the *Open-Field Test* than the L-tailed individuals.

**Figure 4.**
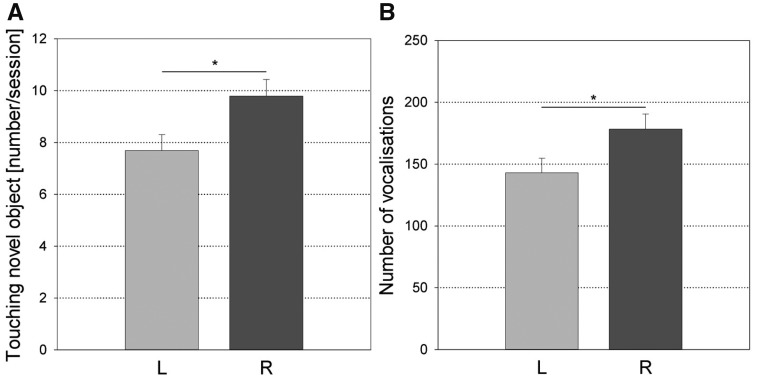
Differences for (**A**) the frequency of touching the Novel Object in the Novel Object Test (*F*_1,55_=5.75, *P *=* *0.020, *N *=* *61) and (**B**) the number of vocalizations in the Open-Field Test (*F*_1,55_= 4.42, *P *=* *0.040, *N *=* *61) between the left- (L) and right-snouted (R) individuals (least square means and standard errors). *P < 0.05.

**Figure 5.**
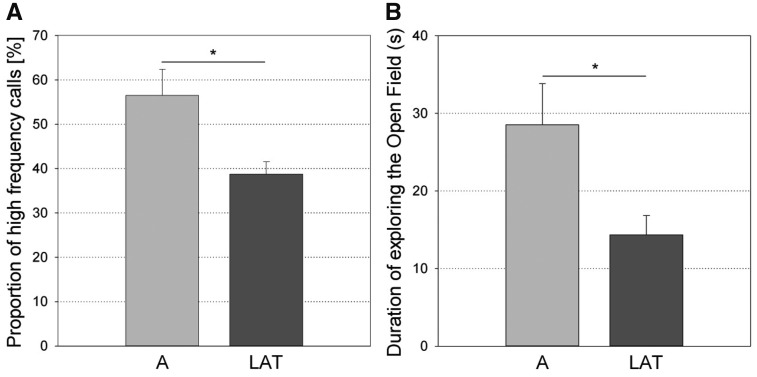
Differences for (**A**) the proportion of high frequency calls produced in the Open-Field Test (*F*_1,70_=6.79, *P *=* *0.011, *N *=* *76) and (**B**) the duration of exploring the Open Field in the Open-Field Test (*F*_1,70_=5.42, *P *=* *0.023, *N *=* *76) between the lateralized (LAT) and ambilateral (A) individuals based on manipulation with the snout (least square means and standard errors). *P <* *0.05.

Concerning the direction of snout laterality (Figure 4), the R-snouted individuals produced more vocalizations (*F*_1,__55_ =4.42, *P *=* *0.040, *N *=* *61, power <0.7) during the *Open-Field Test* and explored the Novel Object more often (*F*_1,__55_ =5.75, *P *=* *0.020, *N *=* *61, power <0.7) during the *Novel Object Test* than the L-snouted individuals. Concerning the strength of snout laterality (Figure 5), the A individuals produced a higher proportion of high frequency calls (*F*_1,__70_ = 6.79, *P* = 0.011, *N* * *= * *76, power = 0.828) and explored the Open Field longer (*F*_1,__70_ = 5.42, *P* = 0.023, *N* = 76, power = 0.738) than the LAT individuals in the *Open-Field Test*.

### Effect of the combined laterality classification on personality parameters

**Figure 6.**
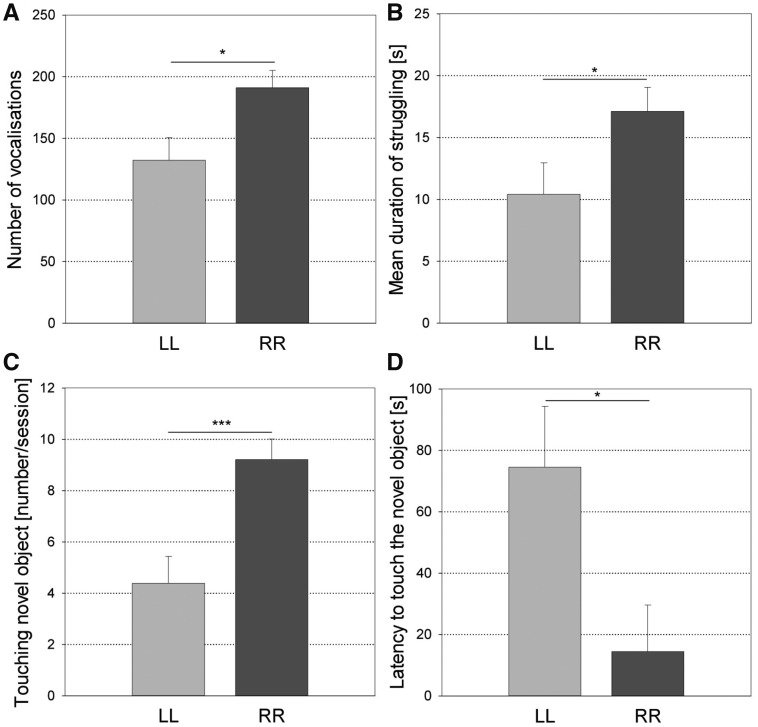
Differences for (**A**) the number of vocalizations in the Open-Field Test (*F*_1,24_ = 7.14, *P *=* *0.013, *N *=* *30), (**B**) the duration of struggling during the Backtest (*F*_1,24_ = 4.90, *P *=* *0.037, *N *=* *30), (**C**) the frequency of touching the Novel Object in the Novel Object Test (*F*_1,24_=14.92, *P *<* *0.001, *N *=* *30), and (**D**) the latency to touch the Novel Object in the Novel Object Test (*F*_1,24_ = 6.38, *P *=* *0.019, *N *=* *30) between left-biased (LL) and right-biased (RR) pigs (least square means and standard errors). *P <* *0.05, ***P <* *0.001.

Concerning the direction of combined laterality (Figure 6), the R-biased individuals vocalized more (*F*_1,__24_ = 7.14, *P* = 0.013, *N* = 30, power = 0.780) during the *Open-Field Test*, and they had a shorter latency (*F*_1,__24_ = 6.38, *P* = 0.019, *N* = 30, power = 0.733) and a higher frequency for touching the Novel Object (*F*_1,__24_ = 14.92, *P* < 0.001, *N* = 30, power = 0.977) during the *Novel Object Test* than the L-biased individuals. In addition, the R-biased individuals struggled longer during the *Backtest* (*F*_1,__24_ = 4.90, *P* = 0.037, *N* = 30, power < 0.7) than the L-biased individuals.

## Discussion

We observed different associations between laterality and personality, depending on the personality trait and the laterality pattern, as well as the measure of laterality (direction or strength; summarized in Table 2). In the following, when discussing each single motor bias, we use the terms of R- or L-snouted or tailed pigs, whereas when describing the combined laterality classification we use the terms R- or L-biased pigs. It appears that the associations depended on the nature of the personality test. Although we found several significant effects of motor laterality on behavior in the *Open-Field Test* and *Novel Object Test*, we did not find any significant effects for the *Human Approach Test* and the *Open Door Test*, which may reflect an effect of group testing (in the *Human Approach Test* and *Open Door Test*) on personality (Lawrence et al. 1991; Koski and Burkart 2015). Additionally, we found no significant effects of motor laterality on the T/C ratio. This is not in line with findings of lateralized effects at the cerebral level in humans (Terburg et al. 2009) although there are—to our knowledge—no findings about a link between this ratio and motor laterality. Moreover, it would be beneficial to further investigate this ratio in pig research and test whether, similarly to humans, interactions with status-relevant behaviors can be found (Mehta and Prasad 2015).

**Table 2.** Summary of the significant effects of motor laterality on the different personality traits: → individuals with a right bias scored higher on the personality trait, ← individuals with a left bias scored higher on the personality trait, ↑ individuals with a strong bias scored higher on the personality trait, ↓ individual with a weak bias scored higher on the personality trait

Laterality	Direction			Strength
Personality	Tail	Snout	Combined	Snout
Boldness	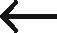		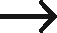	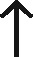
Exploration		→	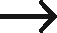	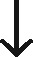
Activity				
Sociability	→	→	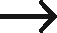	
Aggressiveness				
Coping			→^a^	

Bold arrows indicate results with sufficient power (>0.7)

a R-biased individuals showed more active coping during the *Backtest*.

The direction of the single motor bias (snout use and tail curling) showed significant associations with 2 personality parameters. R-tailed and R-snouted pigs vocalized more in the *Open-Field Test* than L-tailed and L-snouted pigs, which suggests that they were more sociable. R-snouted pigs touched the Novel Object more often in the *Novel Object Test*, suggesting that they were more explorative than L-snouted pigs. However, only one of the significant effects of the direction of the single motor biases on the personality traits reached a sufficient level of power, suggesting that most of these results are not very robust. R-tailed pigs produced a greater proportion of high frequency calls than the L-tailed pigs, indicating that R-tailed pigs were less bold. This seems to contradict findings in other species, where a L bias was associated with increased fearfulness (Hopkins and Bennett 1994; Braccini and Caine 2009). However, Gordon and Rogers (2010) found that R handed marmosets also produced more mobbing or alarm calls in a threatening context. The authors discussed that mobbing calls may not only express fear, but are also used to recruit conspecifics (Clara et al. 2008) and therefore interpreted them as an indicator of proactive behavior. Similarly, although in young pigs the high frequency call proportion has been found to reflect fear during isolation (Leliveld et al. 2016) they may also function to recruit adult sows (Weary and Fraser 1995; Weary et al. 1996).

The strength of laterality could only be analyzed for snout use, where it showed 2 significant associations with Boldness and Exploration. Ambilateral pigs produced a higher proportion of high frequency calls in the *Open-Field Test* than lateralized pigs, suggesting they were less bold (but see discussion above). This is consistent with the findings of reduced Boldness in ambilateral cats and dogs (Branson and Rogers 2006; McDowell et al. 2016) but in contradiction with the findings of increased boldness in ambilateral elks (Found and St. Clair 2017). Ambilateral pigs also explored the Open Field longer during the *Open-Field Test*. To our knowledge, this is a first report of a link between exploration and strength of laterality. Both these results had sufficient power and support the idea that strength of laterality can show different associations with different personality traits, like it has been shown for Playfulness, Aggressiveness, and Sociability in dogs (Barnard et al. 2017).

In contrast to the associations with single motor biases, 3 of the significant associations with the combined laterality classification had sufficient power. R-biased individuals touched the Novel Object more often in the *Novel Object Test*, suggesting they were more explorative, which is consistent with previous studies; R-handed primates explore novel items more than L-handed primates (Cameron and Rogers 1999; Braccini and Caine 2009). In addition, R-biased pigs had a shorter latency to touch the Novel Object, suggesting that they were bolder, which is also consistent with previous studies (Hopkins and Bennett 1994; Cameron and Rogers 1999; Wright et al. 2004; Braccini and Caine 2009; Gordon and Rogers 2010). Our results on Boldness and Exploration are consistent with the approach-withdrawal hypothesis formulated by Davidson (1992), which also appears to be supported by findings in other non-human species (reviewed in Rogers 2010): individuals with a supposed left hemispheric dominance—R-biased individuals—approached more quickly and explored more actively in the context of novelty than individuals with a supposed right hemispheric dominance—L-biased individuals. Additionally, R-biased pigs also vocalized more in the *Open-Field Test*, which may indicate a stronger motivation to regain contact with group members (Murphy et al. 2014; Koolhaas and van Reenen 2016) and therefore indicate greater Sociability. Alternatively, other authors have suggested (Manteuffel et al. 2004) that the call rate (number of vocalisations) of pigs during the *Open-Field Test*, a context of social isolation, can also be used as an indicator of Fearfulness—that is, lower Boldness according to the framework of Réale et al. (2007). However, the classification into call types may provide more insight into pig personality or emotionality than the call rate only (Friel et al. 2016; Leliveld et al. 2016, 2017). Because no associations were found with the proportion of high frequency calls, it seems more likely that the R-biased subjects were not more fearful but more sociable than the L-biased subjects. Therefore, this result appears to be consistent with previous findings of greater Sociability in R-handed primates (Westergaard et al. 2003; Gordon and Rogers 2010). We found that R-biased pigs struggled longer than L-biased pigs, which suggests a more active coping style (Zebunke et al. 2015, 2017). This would be consistent with the hypothesis that the left hemisphere controls proactive behavior, whereas the right hemisphere controls reactive behavior (Rogers 2009, 2010). However, we remain cautious in the interpretation of this result because its power was not high and no effects on the other *Backtest* parameters (latency and frequency) could be tested because of significant interactions between the replicate and the combined laterality classification. Taken together, our results support the approach-withdrawal hypothesis: R-biased pigs approached the Novel Object more quickly and more often than L-biased pigs. They also support previous findings in marmosets that suggest that R-handed individuals are more sociable: R-biased pigs were more vocally active than L-biased pigs. This first evidence of complex links between personality and laterality in pigs indicates that the neurophysiological processes underlying individuality (Freund et al. 2013) are shared between the 2.

Comparing the single motor biases (tail, snout) and the combined laterality classification, we found that the single motor biases showed weak and often varying associations with the personality traits, while the combined laterality classification showed more robust associations. This is in line with previous findings in dogs and chimpanzees that showed different links with personality depending on the motor function (Hopkins and Bard 1993; Batt et al. 2009; Barnard et al. 2017), as well as studies in different species that showed that individual hemispheric dominance does not automatically lead to a consistent direction for each individual motor function (Noonan and Axelrod 1989; Laska 1998; Mohr et al. 2003). By combining the 2 motor biases, we expected to provide insight into individual hemispheric dominance, which is suggested to result in individual behavioral patterns (Rogers 2009; Wright and Hardie 2015; Goursot et al. 2017). Indeed, the combined laterality classification showed robust associations with 3 personality traits, suggesting that this may be a good approach to the multifactorial nature of laterality and a good alternative to other more demanding approaches (e.g., fMRI) that could be otherwise used to determine the cerebral processing that may underlie differences in hemispheric dominance (Mazoyer et al. 2014) and personality.

To summarize, our study suggests that taking the multifactorial nature of laterality and personality into account will contribute to a better understanding of individuality. Both of these aspects of individuality have implications for the welfare of animals under human care, as they may influence how an animal perceives and evaluates its environment.

## Ethical Statement

The experimental procedure was approved by the ethics committee of the federal state of Mecklenburg-Western Pomerania, Germany (LALLF M-V/TSD/77221.3-2-040/14-1) and adhered to the legal requirements of European Union (directive 2010/63/EU).

## Author Contributions

All authors contributed to the planning of the experiments, the data evaluation, and interpretation as well as the preparation of the manuscript. C.G. and L.M.C.L. performed the experiments.

## Supplementary Material

zoy071_Supplementary_Data
